# Clinical outcome of the patients with femoropopliteal artery disease after endovascular therapy: focused on drug-coated-balloon-related distal embolism detected by laser doppler flowmetry

**DOI:** 10.1007/s12928-021-00815-1

**Published:** 2021-10-19

**Authors:** Kuniyoshi Fukai, Takuo Nakagami, Tetsuro Hamaoka, Masakazu Kikai, Shinichiro Yamaguchi

**Affiliations:** 1 Department of Cardiology, Omihachiman Community Medical Center, Omihachiman, Shiga Japan; 2 Department of Cardiology, Japanese Red Cross Kyoto Daini Hospital, Kyoto, Japan; 3 Department of Cardiology, Rakuwakai Marutamachi Hospital, Kyoto, Japan

**Keywords:** Peripheral artery disease, Drug-coated balloon, Embolism, Laser-doppler flowmetry

## Abstract

Several trials have shown that paclitaxel drug-coated balloons (DCBs) significantly reduce restenosis rates. However, some reports have shown distal embolisms occurring after DCBs. No study has analyzed the clinical outcomes of patients with DCB-induced distal embolism. This study aimed to investigate the clinical outcomes of DCB-induced distal embolism in patients with femoropopliteal artery disease. Between February 2018 and April 2019, consecutive patients (*n* = 32) who presented with de novo femoropopliteal artery disease and underwent endovascular therapy using DCB were retrospectively reviewed in a single-center study. Patients were divided into two groups based on whether distal embolism was detected using laser doppler flowmetry (DEL group) or not (non-DEL group). Baseline characteristics and 1-year clinical outcomes were compared between the groups. DEL was found in 44% of limbs (DEL group: *n* = 15, non-DEL group: *n* = 19). Below-the-knee arterial runoff ≤ 1 (*p* = 0.033), popliteal lesion (*p* = 0.044), ambulation difficulty (*p* = 0.021), and previous history of coronary artery disease (*p* = 0.013) were identified as predictive factors of DEL**.** Procedural factors, reference vessel diameter, lesion length, and total drug amount were not predictive of DEL. The overall target lesion restenosis (TLR) rate was 17.4% (*n* = 5). The TLR rate was not significantly different between the DEL and non-DEL groups (13.3% vs. 15.8%, *p* = 0.55). Severe calcification was the only significant factor for TLR (4.2% vs. 40.0%, *p* = 0.02). Among patients with femoropopliteal disease, there was no difference in 1-year clinical outcome between patients who underwent DEL and those who did not.

## Introduction

Endovascular therapy (EVT) is widely considered an effective treatment for symptomatic femoropopliteal artery disease. In recent years, drug-coated balloons (DCBs) have been mainly used in interventions of the femoropopliteal artery. Some clinical trials on DCB showed higher patency rate than conventional balloon angioplasty [1–3]. However, various complications, including distal embolism [4], downstream panniculitis [5], vasculitis [6], and acute hypersensitivity reactions [7] have been reported in studies on DCB. Therefore this study aimed to investigate the clinical outcome of distal embolism associated with DCB use and identify clinical predictors of the occurrence of distal embolism, based on the characteristics of lesions in the femoropopliteal area and patient background.

## Methods

### Study design

**Fig. 1:**
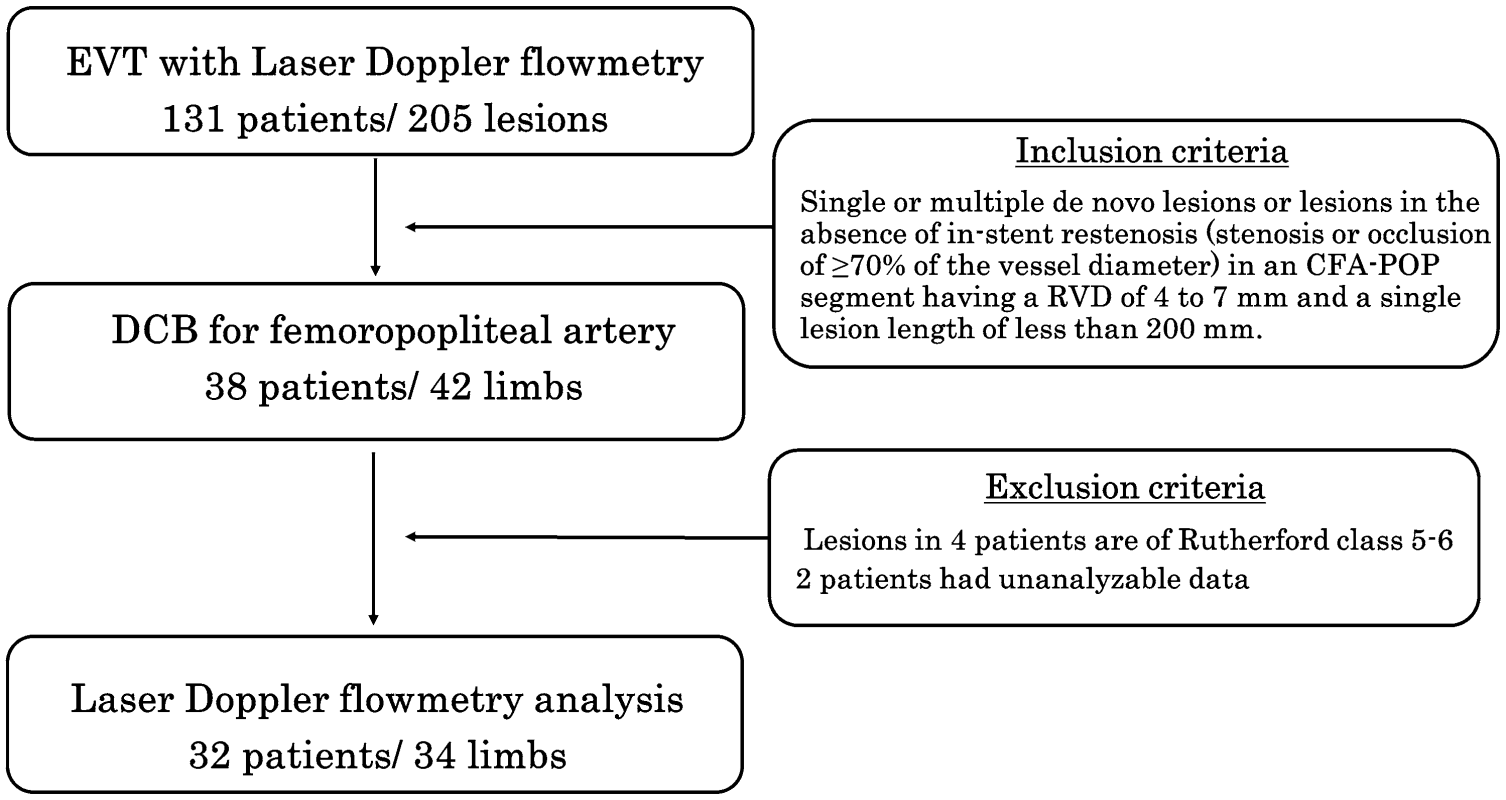
Flow chart showing patient and lesion characteristics. *EVT* endovascular therapy, *CFA* common femoral artery, *POP* popliteal artery, *RVD* reference vessel diameter, *DCB* drug-coated balloon

This study was a retrospective, single-center clinical investigation. Between February 2018 and April 2019, 131 consecutive patients underwent EVT with toe blood flow monitoring by laser doppler flowmetry at the Omihachiman Community medical center. Of these patients, 38 patients underwent DCB for femoropopliteal artery lesions. Limb ischemia severity in four patients was Rutherford class 5–6, and two patients had unanalyzable laser doppler flowmetry data. These patients were excluded from the study. We enrolled the remaining 32 patients (34 lesions) in our analysis (Fig. 1). IN.PACT™ Admiral™ (Medtronic, Minneapolis, USA) and LUTONIX^®^ (BARD, Murray Hill, NJ, USA), which were commercially available in the Japanese market during the study period, were selected as the DCBs of choice, using Japanese criteria as mentioned below.

IN.PACT Admiral DCB was used for single or multiple de novo lesions or in the absence of in-stent restenosis (stenosis or occlusion of ≥ 70% of the vessel diameter), in a femoropopliteal segment with a reference vessel diameter (RVD) of 4–7 mm and a single lesion length of < 200 mm. If the normal blood vessel between two lesions was 3 cm or more, the lesions were considered to be two independent lesions. Moreover, after pre-plain old balloon angioplasty, the residual stenosis rate is less than 50% and there is no severe dissection (grade D or higher).

LUTONIX DCB was used for single or multiple de novo lesions or in the absence of in-stent restenosis (stenosis or occlusion of ≥ 70% of the vessel diameter), in the segment from the common femoral artery to the popliteal artery with an RVD of 4–6 mm and a single lesion length of < 150 mm. In case of multiple lesions, DCB can be used for a lesion with a length of ≤ 15 cm from the proximal to the distal end. Moreover, after pre-plain old balloon angioplasty, the residual stenosis rate is less than 50% and there is no severe dissection (grade D or higher).

The study protocol, which was according to the stipulations of the Declaration of Helsinki, was approved by the institutional review board. All authors confirm that written consent for the submission and publication of this report, including images and associated text, was obtained from the patients.

### Procedural details

EVT was performed using either a contralateral or ipsilateral femoral access. A bare balloon (12/34, 35.2%) or a scoring balloon (22/34, 64.7%) was used in the patients. The average diameter of the balloon was 5.0 mm.

Seventeen lesions (50%) were treated with IN.PACT Admiral DCB**,** while the remaining 17 lesions were treated with LUTONIX DCB

### Follow-up

Clinical evaluations were performed at 1, 6, and 12 months after EVT. Lesion patency was assessed using duplex ultrasound, and decisions regarding vessel patency was based on the consensual analysis of experienced sonographers at the individual institutions. Primary patency was defined as a resting peak systolic velocity ratio of < 2.5 on duplex ultrasound, without reintervention [8]. Clinically driven target lesion restenosis was defined as cases in which reintervention was performed due to a > 50% vessel stenosis, as identified by duplex ultrasound, with recurrent clinical symptoms. Determining the degree of lesion calcification was according to the definitions of the Peripheral Academic Research Consortium [9]. Angiographic dissection pattern was classified according to definitions in previous reports [10, 11].

### Evaluation of blood flow patterns

**Fig. 2:**
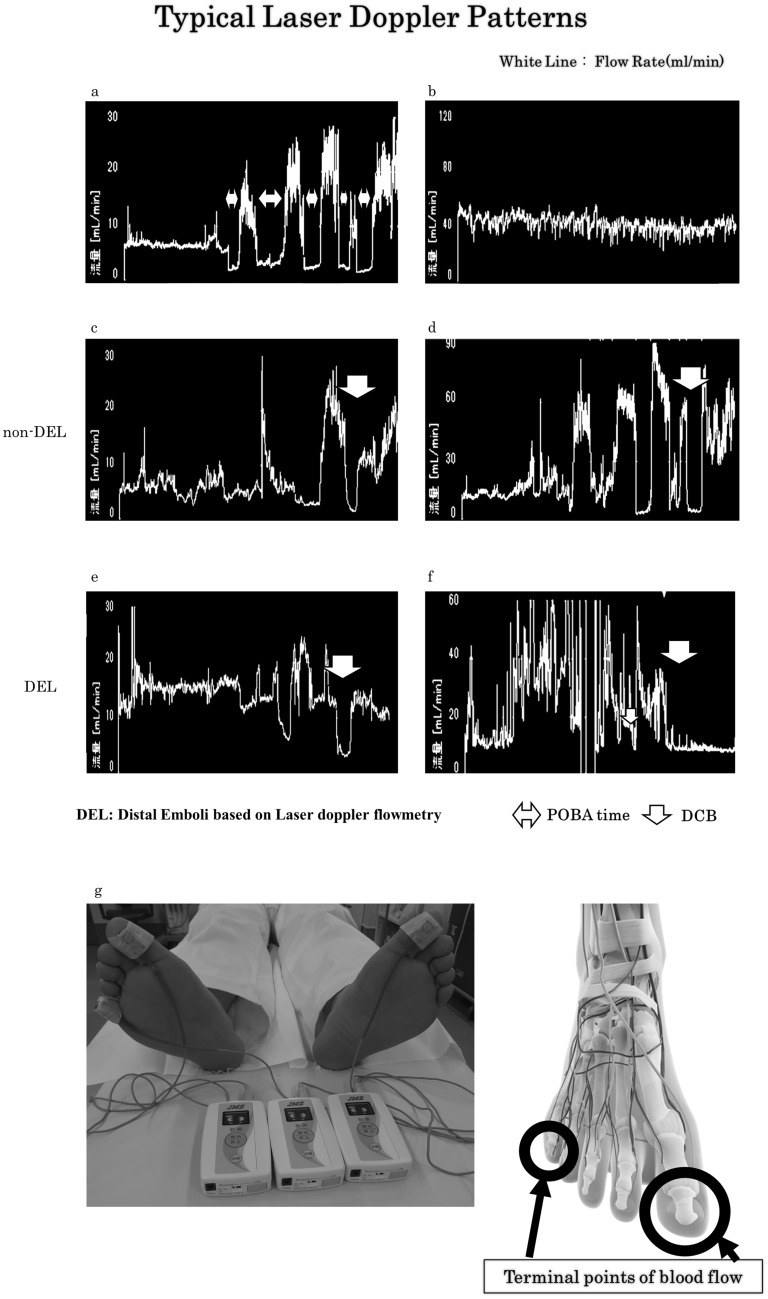
Graphs show typical laser doppler patterns during balloon angioplasty. White line shows flow rate (mL/min). **a**, **c**, **d** Typical improved cases. A drop in POBA time is seen (double arrows). Soon after POBA, flow rate increased soon after POBA. **b** Control data patterns. Flow rates are unchanged or stable. **e**, **f** Typical distal embolism patterns. There are two main patterns: one in which the flow drops gradually (**e**) and one in which the flow drops suddenly (**f**). **g** Attaching the laser doppler device. We chose the first and fifth toes as the terminal points of blood flow. We used the first and fifth toes of the contralateral leg as controls. *POBA* plain old balloon angioplasty

The JMS Pocket LDF^®^ (JMS Co., Ltd., Tokyo, Japan) was used to measure tissue blood flow. Concerning the operation of this device, laser beams are produced by a semi-conductor laser diode that is installed in the LDF probes. These beams penetrate the skin, hit red blood cells in the vasculature, and are dispersed. The laser beams are then converted to scattered light by frequency variation (Doppler shift), which are recognized as electrical signals by a photodetector [12–16]. In this study, a distal embolism event was defined as the occurrence of reduced blood flow in the first or fifth toe around the time of DCB treatment (DEL). Factors that affect flow rate include room temperature, patient’s emotional state, patient’s position, and the activities of the autonomic nervous system. Therefore, it is necessary to continuously measure and monitor the blood flow in the toes of the opposite leg during EVT. Because technically or in physiology, there is no normal value for blood flow in any limb, the therapeutic effect should be evaluated as a relative measure before and after treatment. A good condition is indicated by (1) a clear pulse wave and (2) stable or unchanged control data. Only cases that met the above criteria were analyzed. Based on Laser-doppler flowmetry data, there were two main patterns of distal embolism: a pattern that suggests sudden drop in flow and a pattern that suggests gradual drop in flow (Fig. 2).

### Statistical analysis

Continuous variables are presented as median (interquartile range). Categorical variables are presented as count (percentage). The Wilcoxon signed-rank test was used to compare continuous variables, and the chi-square test was used for categorical variables, as appropriate. Univariate and multivariate logistic regression analyses were used to investigate the independent predictors of restenosis. Clinical variables considered to contribute to restenosis in previous reports were included in the multivariate analysis. Statistical significance was defined as *p* < 0.05. All statistical analyses were performed using JMP software (version 14.2.0, SAS Institute, Cary, North Carolina, USA).

## Results

Fifteen (44%) patients experienced DEL event. DEL appeared in eight cases in IN.PACT Admiral DCB groups and seven cases in LUTONIX groups. Baseline patient characteristics are summarized in Table 1. The mean age was 73.4 years, and 22 patients (64.7%) were men. Further, 88% of patients had hypertension, > 50% had diabetes, and > 25% were smokers. There were significant differences in patient baseline characteristics and the proportion of patients with a previous history of coronary artery disease (93.5% vs. 57.8%; *p* = 0.013) and ambulation difficulty (20% vs. 0%; *p* = 0.021) between the DEL (*n* = 15) and non-DEL (*n* = 17) groups. Baseline lesion characteristics are shown in Table 2. As categorized by the Rutherford classification, the severity of limb ischemia ranged from class 2 to 4. The mean lesion length at baseline was 109 ± 79.0 mm. There were 11 (32.3%) chronic total occlusions. Six (27.2%) lesions were classified as TransAtlantic Inter-Society Consensus class C or D. Below-the-knee arterial runoff ≤ 1 vessel (33.3% vs 5.26%; *p* = 0.033) or the presence of popliteal lesions (40% vs 10.5%; *p* = 0.044) were significantly higher in the DEL group than in the non-DEL group. Other procedural factors, including RVD, lesion length, DCB diameter, and total DCB length were not significantly different between the groups. Regarding clinical outcome, the restenosis or occlusion rate was 17.4% (*n* = 4). Severe calcification was the only factor that influenced the 12-month outcome. DEL was not significantly different between the groups (Table 3). No deaths were recorded in both patient groups.

**Table 1:** Baseline patient characteristics

	Overall (*n* = 34)	DEL (*n* = 15)	Non-DEL (*n* = 19)	*p* value
Age, years	72 (67.75–80.25)	75.2 ± 9.1	72.0 ± 7.7	0.21
Men	22 (64.7%)	12 (80%)	10 (52.6%)	0.09
Hypertension	30 (88.2%)	12 (80%)	18 (94.7%)	0.18
Diabetes	18 (52.9%)	10 (66.6%)	8 (42.1%)	0.15
Dyslipidemia	26 (76.4%)	11 (73.3%)	15 (78.9%)	0.70
BMI	22.39 (20.37–26.01)	22.6 ± 3.32	22.7 ± 3.77	0.77
Current smoker	10 (33.3%)	5 (38.4%)	5 (29.4%)	0.94
Hemodialysis	5 (14.7%)	2 (13.3%)	3 (15.7%)	0.84
Coronary artery disease	25 (73.5%)	14 (93.3%)	11 (57.8%)	0.01
Myocardial infarction	4 (11.7%)	2 (13.3%)	2 (11.1%)	0.49
Cerebrovascular disease	8 (23.5%)	2 (13.3%)	6 (31.5%)	0.20
LVEF < 40%	4 (11.7%)	2 (13.3%)	2 (11.1%)	0.80
eGFR	54.15 (29.5–69.45)	54.7 (39.6–81.6)	51.2 (12.3–67.5)	0.41
Rutherford classification				
Category 2/3	32 (94.1%)	13 (86.6%)	19 (100%)	
Category 4	2 (5.9%)	2 (13.3%)	0 (0%)	0.06
Cilostazol	6 (18.1%)	4 (26.6%)	2 (11.1%)	0.20
Dual antiplatelet therapy	22 (66.6%)	11 (73.3%)	11 (61.1%)	0.45
DOAC	4 (11.7%)	2 (13.3%)	2 (11.1%)	0.80
Warfarin	3 (9.1%)	1 (6.8%)	2 (11.1%)	0.65
Statin	24 (72.7%)	13 (86.6%)	11 (61.1%)	0.18
*Ambulation difficulty	3 (8.8%)	3 (20.0%)	0 (0%)	0.02

The continuous data are shown as median (interquartile range). The categorical data are expressed as mean (SD) or *n* (%)

BMI = body mass index; DOAC = direct oral anticoagulant; eGFR = estimated glomerular filtration rate; LVEF = left ventricular ejection fraction

*Ambulation difficulty means that the patient could not walk for 6 min at a time

**Table 2:** Baseline lesion characteristics

	Overall (*n* = 34)	DEL (*n* = 15)	Non-DEL (*n* = 19)	*p* value
TASC C/D lesions	6 (27.2%)	0 (0%)	6 (31%)	0.25
Lesion length, mm	90 (40–153)	90 (30–160)	90 (70–130)	0.76
RVD, mm	5.1 (4.375–5.65)	5.1 (4.5–6.0)	5.1 (4.3–5.5)	0.48
Occlusions	11 (32.3%)	5 (33.3%)	6 (31.5%)	0.91
Calcification				
Mild or less	20 (58.8%)	8 (53.3%)	12 (63.1%)	
Moderate	5 (14.7%)	3 (20.0%)	2 (11.1%)	0.68
Severe	9 (26.4%)	4 (26.6%)	5 (26.3%)	0.72
BK run off ≤ 1	6 (17.6%)	5 (33.3%)	1 (5.3%)	0.03
Cutting/scoring	22 (64.7%)	10 (66.6%)	12 (63.1%)	0.83
Balloon size, mm	5.0 (4.0–6.0)	5.0 (5.0–6.0)	5.0 (4.0–6.0)	0.48
Total balloon length, mm	120 (60–200)	120 (60–180)	150 (100–200)	0.34
Dissection grade D after DCB	5 (14.7%)	2 (13.3%)	3 (15.7%)	0.84
POP lesion	8 (23.5%)	6 (40.0%)	2 (11.1%)	0.04
Drug amount, μg	4700 (2761–10,622)	4700 (2300–10,200)	4700 (2848–12,254)	0.53

The categorical data are expressed as mean (SD) or *n* (%). The continuous data are shown as median (interquartile range)

Scoring means Lacrosse NSE PTA^®^ (NIPRO, Osaka, Japan) or Scoreflex^®^ PTA (OrbusNeich, Hong Kong, China)

Determining the degree of lesion calcification was according to the definitions of the Peripheral Academic Research Consortium

*RVD* reference vessel diameter, *BK* below the knee, *DCB* drug-coated balloon, *POP* popliteal artery

**Table 3:** Predictive factors for target lesion restenosis

	Odds ratio	95% confidence interval	*p* value
Distal embolism event	0.82	0.09–5.68	0.84
Severe calcification	15.29	1.86–331.99	0.01
Total lesion length ≥ 150 mm	2.09	0.23–15.35	0.47
RVD ≤ 5 mm	1.20	0.30–4.78	0.79
Rutherford classification Category 4	7.00	0.24–203.60	0.22
CTO	1.48	0.17–10.52	0.69
BK run off ≤ 1 vessel	1.19	0.06–10.63	0.88
DM	1.84	0.26–15.65	0.53
Female	1.26	0.14–8.91	0.81

Values are presented as mean (SD), *n* (%), or median (interquartile range). P values were obtained from the chi-square test (for discrete variables) and the Welch t-test (for continuous variables)

Severe calcification is a significant restenosis factor. Other factors did not show a significant difference between the groups

## Discussion

The main finding of this study is the identification of patients at high risk for DCB-induced distal embolism. To the best of our knowledge, this is the first study to report the relationship between 1-year patency rate and DCB-induced distal embolism. DCB use does not affect the clinical outcome of patients with simple femoropopliteal (FP) lesions. Measuring the blood flow of the toe is one of the best clinical methods for detecting distal embolisms. It is difficult to detect distal embolism with angiography alone because angiography is based on visual judgment, and the findings depend on the method of contrast agent administration and the amount of contrast. Using laser Doppler flowmetry, it was possible to accurately capture and analyze distal embolisms. In an experimental model, Torii et al. [17] assessed four groups of treated (percutaneous transluminal angioplasty [PTA] + drug eluting stent (DES), DCB + DES, DCB + bare metal stent [BMS], and DCB alone) iliofemoral arteries of 12 healthy swine, and downstream vascular changes were exclusively observed in the arteries that were treated with DCBs. Kolodgie et al. [18] reported that there was more fibrinoid necrosis in tissues that were treated with IN.PACT DCBs than in those that were treated with LUTONIX DCBs, suggesting increased emboli debris with higher paclitaxel levels. In clinical practice, we found that distal embolism events depend on patient background, such as the degree of patency in the below-the-knee artery or the patient’s gait, and does not depend on the amount of drug administered. Therefore, the decrease in the blood flow of the toe after DCB treatment was not directly linked to clinical outcomes. However, our results may have been affected by our patient selection in which patients with simple FP lesions were included and patients with chronic limb-threatening ischemia who had ulcers or gangrene were excluded. In our opinion, patients with a low vascular bed are more likely to experience blood flow loss after DCB. The limitations of our study are that it is a retrospective study and the study population was small. Therefore, further prospective investigation is needed to evaluate distal embolisms in all patient groups, including those with chronic limb-threatening ischemia [19].

## Conclusion

We evaluated DCB-induced distal embolism using laser Doppler flowmetry. It did not affect the clinical course of patients with simple femoropopliteal disease.
